# Optimization and characterization of biosurfactant production from marine *Vibrio* sp. strain 3B-2

**DOI:** 10.3389/fmicb.2015.00976

**Published:** 2015-09-23

**Authors:** Xiaoke Hu, Caixia Wang, Peng Wang

**Affiliations:** ^1^Key Laboratory of Coastal Biology and Bioresource Utilization, Yantai Institute of Coastal Zone Research, Chinese Academy of SciencesYantai, China; ^2^University of Chinese Academy of SciencesBeijing, China; ^3^Ocean University of ChinaQingdao, China

**Keywords:** microbial biosurfactant, optimal production, surface tension, *Vibrio alginolyticus*, glycoproteins

## Abstract

A biosurfactant-producing bacterium, designated 3B-2, was isolated from marine sediment and identified as *Vibrio* sp. by 16S rRNA gene sequencing. The culture medium composition was optimized to increase the capability of 3B-2 for producing biosurfactant. The produced biosurfactant was characterized in terms of protein concentration, surface tension, and oil-displacement efficiency. The optimal medium for biosurfactant production contained: 0.5% lactose, 1.1% yeast extract, 2% sodium chloride, and 0.1% disodium hydrogen phosphate. Under optimal conditions (28°C), the surface tension of crude biosurfactant could be reduced to 41 from 71.5 mN/m (water), while its protein concentration was increased to up to 6.5 g/L and the oil displacement efficiency was improved dramatically at 6.5 cm. Two glycoprotein fractions with the molecular masses of 22 and 40 kDa were purified from the biosurfactant, which held great potential for applications in microbial enhanced oil recovery and bioremediation.

## Introduction

Oil contamination is a common problem in tanker spills and drilling rig blowouts, which seriously impacts the ecological balance of marine ecosystems (Jernelöv, 2010). Traditional remediation deals with oil contamination by chemical and physical means. Chemical remediation involves the use of chemical compounds as oxidant, reductant, polymer, and/or precipitant (Xue et al., 2015). Physical remediation removes residual oil using adsorbent (Daifullah and Girgis, 2003). The chemical and physical methods are expensive and complex operational, leaving non-biodegradable residues potentially toxic to the environment (Makkar and Cameotra, 2002). Bioremediation uses naturally occurring microorganisms to degrade contaminants and this biological means has been proved to be effective for removing oil contaminants (Rathore et al., 2013).

Hydrocarbons have low solubility and high hydrophobicity, and thus are often unavailable to microorganisms. Biosurfactant-producing microorganisms such as *Pseudomonas aeruginosa* are capable of degrading hydrocarbons and use them as carbon sources (Zhang et al., 2012). Another example is *Vibrio* sp. P-2P44T utilizing polycyclic aromatic hydrocarbons as substrate, such as naphthalene, 2-methylnaphthalene, and phenanthrene (Hedlund and Staley, 2001). Biosurfactants are amphipathic molecules consisting of both hydrophilic and hydrophobic moieties. Such compounds are composed of different biological macromolecules (e.g., lipid, phospholipid, polysaccharide, and protein) and contain various functional groups (e.g., carboxyl, amino, and phosphate; Desai and Banat, 1997).

Surfactants possess functional properties such as detergency, emulsification, foaming, and dispersion (Lee et al., 2008). Microorganisms produce a variety of surfactants, including low-molecular-weight polymers that can efficiently reduce surface and interfacial tension, and high-molecular-weight molecules that act as highly efficient emulsifiers (Toledo et al., 2008). Compared with chemical surfactants, biosurfactants exhibit the advantages of mild production condition, lower toxicity, higher biodegradability, and environmental compatibility (Makkar and Cameotra, 2002). Biosurfactants hold great potential for applications in the environmental protection as well as in the food, cosmetic, biopesticide, and pharmaceutical industries (Singh and Cameotra, 2004).

Thus far, only a few biosurfactants have been produced at large scale for commercial applications, mainly because of their considerable costs of production and recovery (Shete et al., 2006). The controlling parameters of biosurfactant production are required to be maintained within a certain range of operating conditions whereby the activity of bacteria with the resultant maximum production of biosurfactant can be optimized. In this regard, temperature, pH, medium composition, and salinity are of prime importance for controlling and optimizing biosurfactant production (Najafi et al., 2010). *Pseudomonas, Candida*, and *Rhodococcus* have been studied extensively regarding surfactant biosynthesis (Singh et al., 1990; Espuny et al., 1996). Currently, relative little information is available on the optimal conditions for biosurfactant production from *Vibrio* species.

If maintained under optimal conditions for growth and activity, that biosurfactant producer can only be improved. Response surface methodology (RSM) is one of the best methods to design the optimization experiments (Wei et al., 1998). RSM comprises a set of statistical techniques, which aims to build models, evaluate the effects of operating factors and search for the optimal conditions using design of experiments (Rodrigues et al., 2006). The analytical hierarchy process (AHP) is a technique for organizing the information and judgment used in making complex decisions. The AHP can solve especially where multiple factors and choices (or alternatives) have to be considered simultaneously (Mansora et al., 2013). Application of the AHP has been noted in different industries such as investment appraisal (Hedlund and Staley, 2001), project selection and human resource evaluation. No study has reported the use of the AHP in medium optimization.

In this study, a biosurfactant-producing *Vibrio* strain, designated 3B-2, was isolated from marine sediment. The objectives of this study were: (1) to identify the physical and chemical factors affecting biosurfactant production from strain 3B-2, and (2) to find the optimal composition of growth medium for flask-scale biosurfactant production using the RSM and AHP techniques. The results may provide hints at potential applications of biosurfactant from marine *Vibrio* in environmental, biomedical and agricultural fields.

## Materials and Methods

### Isolation and Characterization of Biosurfactant-Producing Bacteria

Fresh sediment samples (10 g) and crude oil (1 g) were co-incubated in 100 mL of mineral medium in darkness for 7 days, with shaking at 180 rpm at 30°C. The mineral medium contained: 7.01 mM K_2_HPO_4_, 2.94 mM KH_2_PO_4_, 0.81 mM MgSO_4_⋅7H_2_O, 0.18 mM CaCl_2_, and 1.71 mM NaCl (Lin et al., 2014). The pH of the medium was adjusted to 7.0 using 1 M NaOH. Crude oil used in this study was obtained from the Shengli Oil Field, Dongying, Shandong, China, and sediment was collected from the Bohai Bay, an area adjacent to Penglai 19-3 oil platform (120°01′∼120°08′E, 38°17′∼38°27′N), which were kindly supplied by the North China Sea Branch of The State Oceanic Administration. For both sediment and oil samples, we collected three tubes (∼50 g) from different positions of a bucket to form a composite sample. To isolate bacterial strains, zero point one-milliliter aliquots of the sediment culture were aseptically spread onto Marine Agar 2216E plates (Difco, USA). The inoculated plates were incubated at 30°C for 48 h. Morphologically distinct colonies were obtained by repetitive streaking five times onto 2216E plates.

Surface tension and the diameter of clear zone on the oil surface were measured to evaluate the production of biosurfactant and screen the isolates of interest. The lower the surface tension, the better the biosurfactant; the diameter of clear zone depends on the concentration of the biosurfactant (Youssef et al., 2004). A strain with rapid growth and higher biosurfactant production was selected and named 3B-2. Purified culture samples of 3B-2 were dispensed into cryovials, added with 15% glycerol, and preserved at -20°C immediately. Strain 3B-2 was examined by morphological observations which the Gram staining was tested with photomicroscopy, morphological trait of the isolated strain was observed under a scanning electron microscope. Phenotypic analysis and various biochemical tests were carried out by following what was described in Bergey’s Manual of Systematic Bacteriology (Noel and George, 2005).

### 16S rDNA Sequencing

Bacterial genomic DNA was extracted using a DNA extraction kit (Sigma, USA) following the manufacturer’s instructions. PCR amplification of bacterial 16S rDNA was performed using universal primers 27F (*5*′*-AGAGTTTGATCMTGGCTCAG-3*′) and 1492R (*5*′*-CGGYTACCTTGTTACGACTT-3*′) as previously described (Enticknap et al., 2006). The PCR product of 16S rDNA from strain 3B-2 (1119 bp) was directly sequenced by Sangon Biotech (Shanghai) Co., Ltd. The obtained sequence was compared using the BLAST Tool of NCBI^1^. Closely related sequences were retrieved from the GenBank database and sequence alignment was performed using ClustalW v2.0. Phylogenetic tree was constructed using the maximum-likelihood algorithm in MEAG v5.0^2^.

### Biosurfactant Production and Purification

Strain 3B-2 was transferred into 5 mL of modified Marine Broth 2216E (0.1% yeast extract, 0.5% peptone, and 1.5% NaCl) and incubated for 24 h (32°C, 180 rpm) to obtain the seed culture. Then 5 mL of culture suspensions were transferred to a 250-ml Erlenmeyer flask containing 100 mL of modified 2216E. The culture was incubated with shaking at 180 rpm, 32°C for 48 h. Thereafter, the supernatant was removed from the culture broth by centrifugation at 8,000 rpm, 4°C for 10 min. The collected cells were re-suspended in 10 mL of distilled water and crude biosurfactant was extracted using an ultrasonic cell disintegrator (JY 92-IID, Xinzhi Biotech., Ningbo, China). The extraction conditions were as follows: amplitude transformer Φ10, power 70% and extraction time 30 min. Then the ultrasonic extract was centrifuged at 7000 rpm for 10 min at 4°C. The supernatant was used as the crude biosurfactant and preserved at -20°C before analysis.

The AKTA protein purification system was used to purify crude biosurfactant. The sample was applied to a HiPrep DEAE-FF column (1.0 cm × 1.0 cm) pre-equilibrated with ultra-pure water (mobile phase A), and then eluted using a gradient of buffer from 0 to 1 M (1 M NaCl, mobile phase B) at a flow rate of 1.0 mL/min. Proteins were detected at 280 nm. Fractions exhibiting oil-displacement activity were pooled, concentrated, and injected into a Sephrcryl S-100 HR column (1.0 cm × 80 cm) equilibrated with ultra-pure water at 0.3 mL/min. Further, fractions were collected and screened for oil-displacement activity.

### Analytical Methods

#### Biomass Estimation

Biomass was monitored by spectrophotometry (Martinez et al., 2000). The optical density (OD) of culture samples at 600 nm wavelength was measured and the blank medium was adjusted to OD_600_ = 0.

#### Protein Concentration

The protein level of crude biosurfactant was determined using Lowry’s method (Lowry et al., 1951) with bovine serum albumin (Sigma, America) as a protein standard.

#### Surface Tension Measurement

The surface tension of crude biosurfactant was measured using a digital surface tensiometer (BZY-201, Fangrui, Shanghai, China) by the Du Nüoy ring method (Santos et al., 2014). Briefly, 10 mL of samples were transferred into a clean plate and placed on the tensionmeter platform. A platinum strip was slowly put on the liquid-air interface to measure surface tension (mN/m). The platinum strip was washed (with chromic acid, ultrapure water, and acetone), flamed and dried between each measurement. Calibration was performed using ultrapure distilled water (surface tension = 71.5 ± 0.5 mN/m) before sample measurement.

#### Oil Displacement Test

The procedure of oil displacement test for surfactants was slightly modified from a previously reported method (Morikawa et al., 1993). Firstly, 1 g of crude oil (Shengli Oil Field) was dissolved in 20 mL of chloroform to obtain an oil solution. Secondly, 2 mL of oil solution was put onto the surface of 20 mL of distilled water in a 15-cm diameter Petri dish to form a thin membrane of oil. Then, 40 μL of crude biosurfactant was put on the center of the oil membrane. A clear zone was visible immediately on the oil surface and the diameter was measured to calculate oil displacement.

### Optimization Procedure of Biosurfactant Production

#### Physical Parameters

One loopful of bacterial culture from the preliminary slant medium was transferred aseptically to shake flask medium and incubated for 24 h (80 rpm, 32°C) to obtain the seed culture. The seed was inoculated into Modified Marine Broth 2216E and incubated on a 180-rpm rotary shaker at different temperatures (25, 28, 31, 34, and 37°C). The effects of broth content (50, 75, 100, and 125 mL) and inoculum size (4, 6, 8, and 10%) on biosurfactant production from strain 3B-2 were also studied. Biosurfactant production was evaluated using four indices described in Section “Optimization Procedure of Biosurfactant Production.”

#### Carbon and Nitrogen Sources

Strain 3B-2 was grown in Modified Marine Broth 2216E supplemented with different carbon sources (1%, w/v), including glucose, sucrose, lactose, maltose, and xylose. The effect of different nitrogen sources on biosurfactant production was also studied, including beef extract, peptone, yeast extraction, soybean meal, and corn meal (organic; 0.5%, w/v) and (NH_4_)_2_SO_4_, NH_4_NO_3_, NH_4_Cl, NaNO_3_, and urea (inorganic; 0.3%, w/v).

#### Salinity and Ions

On the basis of optimal carbon and nitrogen sources, different concentrations of NaCl (0, 5, 10, 15, 20, and 30 g/L) were added into Modified Marine Broth 2216E. Additionally, different ions (0.05%, w/v) including ZnSO_4_, Na_2_HPO_4_, CaCl_2_, BaCl_2_, CuSO_4_, MgSO_4_, MnSO_4_, and FeCl_3_ were added. The effects of salinity (NaCl concentration) and ions on biosurfactant production from strain 3B-2 were studied.

#### The AHP Method was Used as a Modeling and Optimization Tool

Analytical hierarchy process is a multi-criteria method that can be used to evaluate the importance of each component of the medium. The AHP model consists of three levels: the top level (goal), the second level (criteria), and the third level (alternatives; Ong et al., 2001). In this study, the goal was the optimal factor; the criteria included the biomass, the protein concentration, the surface tension of biosurfactant, and the diameter of clear zone; the alternatives referred to different operating factors.

##### Structure of the AHP model

###### Step 1

**FIGURE 1:**
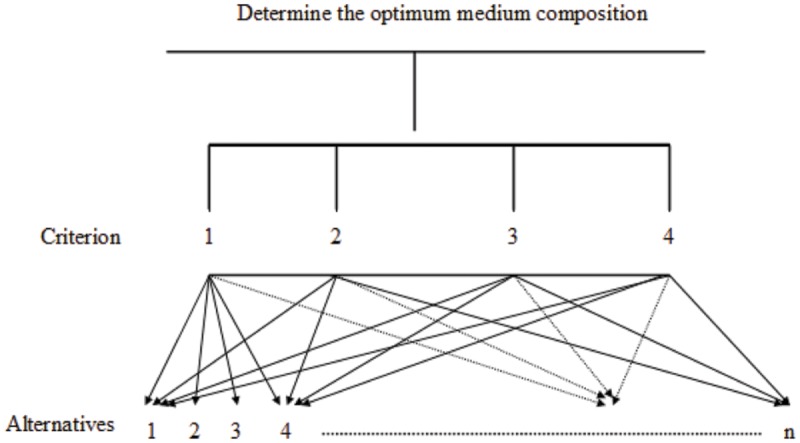
**Optimization of medium composition for biosurfactant production using the analytical hierarchy process**.

In the AHP model, the top level (the goal) is the optimal medium composition (or operating factors). At the second level, there are four criteria: the biomass (OD_600_), the protein concentration, the surface tension, and the diameter of clear zone. At the third level, there are *n* alternatives that must be compared and assessed. An example of the AHP model for a case study of medium composition is shown in **Figure 1**.

###### Step 2

The judgment matrix is structured in each of the three levels. The weights of different criteria are not necessarily the same in the criterion level. The judgment matrix *A = (a_ij_)_n__×__n_* is defined to use discrete 9-point scales (**Table 1**).

**Table 1:** The Saaty fundamental 9-point scale for comparative judgments.

Intensity of importance	Definition
1	Equal importance
3	Moderate importance
5	Strong importance
7	Very strong importance
9	Extreme importance
2, 4, 6, 8	For compromise between above values

###### Step 3

The matrix is normalized and the importance of the criteria is determined using the geometric mean algorithms. The degree of importance for the criteria is set as *w_i_* (Liao, 2010):

(1) $$Wi=\frac{{\left(\Pi_{j=1}^{n}aij\right)}^{1/n}}{\sum_{i=1}^{n}{\left(\Pi_{j=1}^{n}aij\right)}^{1/n}}i,j=1,2,\ldots n$$

###### Step 4

For single hierarchical sorting, a consistency index (*CI*) is determined as follows:

(2) $$CI=\frac{\lambda_{max}-n}{n-1}$$

where *λ_max_* is the maximal eigen value.

A consistency ratio (*CR*) is expressed to check for consistency:

(3) $$CR=\frac{CI}{RI}$$

where *RI* denotes the average random index with the value (**Table 2**). The judgment matrix of consistency is considered reasonable for *CR* < 0.1.

**Table 2:** The random index (*RI*) for consistency check of the judgment matrix.

*N*	1	2	3	4	5	6	7	8	9	10
*RI*	0.00	0.00	0.58	0.90	1.12	1.24	1.32	1.41	1.45	1.49

###### Step 5

The priority for all alternatives is determined and the alternative (supply chain process) with the highest overall priority weight is chosen.

#### Optimization by RSM

Response surface methodology was used to optimize the screening variables for enhanced biosurfactant production using a central composite design (CCD). The significant variables utilized were lactose, yeast extract and NaCl, each at five coded levels (-1.682, -1, 0, +1, and +1.682; **Table 3**). The response values (*Y*) in each trial represent the overall weight.

**Table 3:** The central composite design matrix and experimental results of biosurfactant characterization.

Run	Coded			Test indices				Weight	
	*X*_1_ (lactose)	*X*_2_ (yeast extract)	*X*_3_ (NaCl)	Biomass (OD_600_)	Protein concentration (g/L)	Surface tension (mN/m)	Diameter of clear zone (cm)	Actual value	Predicted value
1	0	0	0	0.59	4.39	37.81	4.30	0.0268	0.0268
2	0	0	-1.682	0.35	2.65	49.36	4.50	0.0161	0.0161
3	1	1	1	0.86	5.29	40.72	5.50	0.0374	0.0374
4	1	-1	-1	0.29	2.29	45.69	4.00	0.0133	0.0133
5	-1	1	-1	0.54	4.37	50.51	6.00	0.0304	0.0304
6	1	1	-1	0.72	5.09	47.57	7.00	0.0409	0.0409
7	0	-1.682	0	0.16	1.32	48.00	0.00	-0.0134	-0.0134
8	-1	-1	-1	0.28	2.02	48.35	0.00	-0.0103	-0.0103
9	0	0	0	0.60	4.11	41.96	5.00	0.0283	0.0283
10	-1	1	1	0.85	5.33	38.25	7.00	0.0466	0.0466
11	1.682	0	0	0.59	4.16	43.38	4.50	0.0251	0.0251
12	1	-1	1	0.37	2.85	41.47	4.50	0.0199	0.0199
13	0	0	0	0.62	4.33	42.89	5.50	0.0315	0.0315
14	0	1.682	0	1.00	6.25	41.36	6.50	0.0467	0.0467
15	0	0	0	0.59	4.50	38.00	4.50	0.0289	0.0289
16	0	0	0	0.59	4.50	38.00	4.50	0.0289	0.0289
17	-1	-1	1	0.36	2.84	43.11	5.00	0.0219	0.0219
18	0	0	1.682	0.67	4.38	37.85	6.00	0.0368	0.0368
19	-1.682	0	0	0.59	4.40	40.36	5.00	0.0296	0.0296
20	0	0	0	0.59	4.50	38.00	4.50	0.0289	0.0289

*OD_*600*_ is the optical density of bacterial culture at 600 nm wavelength.*

### Electrophoretic Analysis

The molecular weight of purified fractions of biosurfactant from 3B-2 was estimated using SDS-PAGE as previously described (Laemmli, 1970).

### Statistical Analysis

The experiments were carried out in triplicate and data were expressed as the mean values. An analysis of variance (ANOVA) for the obtained results was performed, and *p*-values less than 0.05 were considered statistically significant. Design Expert 8.0.6 (Stat-Ease, Inc., USA) was employed for experimental design, ANOVA and process optimization.

## Results and Discussion

### Characterization of Biosurfactant-Producing Strain 3B-2

A total of 20 biosurfactant-producing bacterial strains were isolated from the crude oil-contaminated sediment in Bohai Bay. Among the final candidates, strain 3B-2 was selected for the highest biosurfactant production and oil-displacement activity. The biosurfactant from 3B-2 demonstrated the primary ability of oil displacement at 5.0 cm, and its surface tension dropped below 40 mN/m in Modified Marine Broth 2216E.

Morphological observations revealed that strain 3B-2 formed round, moist, and dense colonies with smooth surface on the screening plates. Detailed biochemical characteristics of 3B-2 are shown in **Table 4**. 3B-2 was a Gram-negative bacterium capable of utilizing glucose, maltose and mannose. It was positive for oxidase activity but negative for O/129. Based on these results, strain 3B-2 was tentatively identified as a member of genus *Vibrio*.

**Table 4:** Physiological and biochemical properties of strain 3B-2 from marine sediment in Bohai Bay, China (VITEK 2 Compact).

Item	Result	Item	Result	Item	Result	Item	Result
APPA	+	ADO	-	PyrA	+	IARL	-
H2S	-	BNAG	+	AGLTp	-	DGLU	+
BGLU	-	dMAL	+	dMAN	+	dMNE	+
ProA	+	LIP	-	PLE	-	TyrA	+
SAC	+	dTAG	-	dTRE	+	CIT	-
ILATK	+	AGLU	-	SUCT	+	NAGA	-
GlyA	+	ODC	-	LDC	+	IHISA	+
O129R	-	GGAA	+	IMLTa	+	ELLM	+
dCEL	-	BGAL	-	GGT	+	OFF	+
BXYL	-	BALap	-	MNT	-	5KG	-
URE	-	DSOR	-	AGAL	-	PHOS	+
CMT	+	BGUR	-	ILATa	-		

### Identification of Biosurfactant-Producing Strain 3B-2

Sequence alignment revealed that 3B-2 was highly related (99% identity) to *Vibrio* species, such as *V. parahaemolyticus* strain H061 (KJ577056.1), *Vibrio alginolyticus* strain B17-4 (KC884590.1), and *V. harveyi* strain NA02 (KJ563265.1). Therefore, strain 3B-2 was identified as *Vibrio* sp.

**FIGURE 2:**
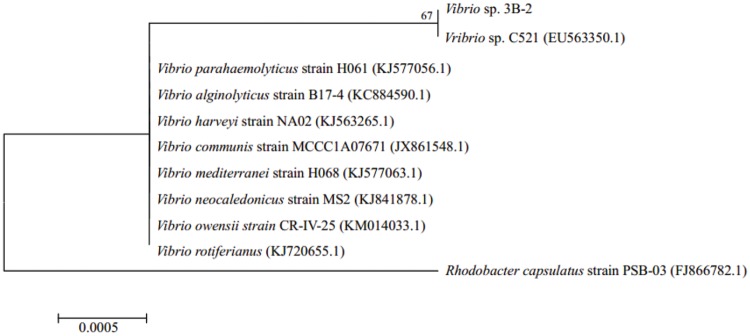
**Maximum-likelihood tree based on 16S rDNA gene sequence of strain 3B-2 isolated from Bohai Bay sediment (1169 bp) and those of known relatives retrieved from the GenBank database (accession No. in brackets).** Bootstrap values shown at nodes for frequencies at or above a 50% threshold (per 1000 trials bootstrap resampling). *Rhodobacter capsulatus* used as outgroup. Bar indicates 0.05% sequence variance.

A phylogenetic tree was constructed based on 16S rDNA sequences of strain 3B-2 and its close relatives retrieved from the GenBank database (**Figure 2**). In the maximum-likelihood tree, 3B-2 was clustered with most known strains of *Vibrio* sp. The result of phylogenetic analysis agreed with those of phenotypic tests. Strain 3B-2 was named *Vibrio* sp. 3B-2 and deposited in the China Center for Type Culture Collection under accession number CJ11052.

### Optimization of Biosurfactant Production

#### Effect of Physical Parameters

Temperature is a critical environmental factor affecting microbial growth and reproduction (Ratkowsky et al., 1982). The effect of temperature on biosurfactant production from strain 3B-2 is shown in **Table 5**. In this test, the goal was the optimal temperature; the alternatives were different temperatures. The matrices were built as described in Section “The AHP Method was used as a Modeling and Optimization Tool.” The *λ_max_* values of the alternatives to the criteria were 5.26, 5.19, 5.22, and 5.22 (*CR* = 0.0585, 0.043, 0.0491, and 0.0481, respectively). The *λ_max_* value of the criteria to the goal was 4.05 (*CR* = 0.017) and that of the alternatives to the goal was 5.22 (*CR* = 0.0485). All the above *CR* values were less than 0.1, which passed the consistency test.

**Table 5:** Effect of temperature on four test indices of biosurfactant production from strain 3B-2.

Temperature (°C)	Biomass (OD_600_)	Protein concentration (g/L)	Surface tension (mN/m)	Diameter of clear zone (cm)	Overall weight
25	0.72	2.72	45.0	4.25	0.1587
28	0.62	2.90	34.6	6.50	0.4998
31	0.65	2.47	41.3	5.75	0.2217
34	0.55	1.82	47.0	4.25	0.0478
37	0.55	2.00	51.1	5.00	0.0720
*λ_max_*	5.2623	5.1926	5.2198	5.2157	5.21728
*CI*	0.0656	0.0481	0.0549	0.0539	0.05432
*RI*	1.12	1.12	1.12	1.12	1.12
*CR*	0.0585	0.043	0.0491	0.0481	0.0485

*λ_max_ = 4.0458, *CI* = 0.0153, *RI* = 0.9, CR = 0.017 (*λ_max_*: the maximal eigen value, *CI*: the consistency index, *RI*: the random index, *CR*: the consistency ratio).*

The obtained *λ_max_* values represent the weights of individual test indices and their overall weight is given in **Table 5**. Results showed that the biosurfactant production from strain 3B-2 gradually increased from 25 to 28°C and the maximal production occurred at 28°C. Increase in the temperature beyond 28°C had an adverse effect on the biosurfactant production (**Table 5**). It has been suggested that microorganisms synthesize only a reduced number of proteins essential for growth and other vital physiological processes at high temperatures (Gawande and Kamat, 1999). For strain 3B-2, the temperature greater than 28°C possibly increased energy consumption and decreased bacterial growth, negatively affecting biosurfactant production. Thus, 28°C was selected for fermentation production of biosurfactant from this bacterium.

**FIGURE 3:**
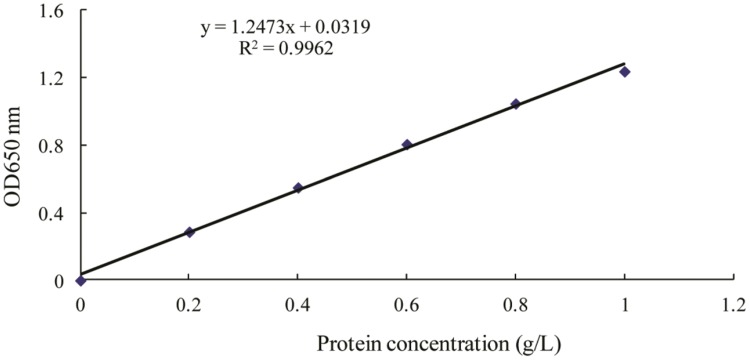
**Standard curve for determination of protein by Lowry’s method (Lowry et al., 1951).** OD_650_ for optical density at 650 nm wavelength.

The changes in biosurfactant production from strain 3B-2 with different liquid volumes, 50, 75, 100, and 125 mL were shown in **Table 6**. Optimization of liquid volume was performed by the AHP based on measurements of the four test indices. In this test, the goal was the optimal liquid volume; the alternatives were different liquid volumes. The *λ_max_* values of the alternatives to the criteria were 4.13, 4.15, 4.16, and 4.13 (*CR* = 0.0497, 0.0563, 0.0586, and 0.0468, respectively). Comparative matrices were built with the biomass, the protein concentration (**Figure 3**), the surface tension, and the diameter of clear zone to the optimal liquid volume as 1, 2, 3, and 4; the *λ_max_* value of the criteria to the goal was 4.05 (*CR* = 0.017); the individual weights of the four criteria to the goal were 0.1, 0.18, 0.29, and 0.43, respectively. The *λ_max_* value of the alternatives to the goal was 4.14 (*CR* = 0.0522). All these *CR* values were less than 0.1 and thus passed the consistency test. The obtained *λ_max_* values represent the weights of individual indices from which the overall weight could be obtained. According to the overall weight, the four test indices were highest with the liquid volume of 100 mL. Liquid volume above or below the optimal level had a negative effect on biosurfactant production from 3B-2 (**Table 6**).

**Table 6:** Effect of liquid volume on four indices of biosurfactant production from strain 3B-2.

Broth content (mL)	Biomass (OD_600_)	Protein concentration (g/L)	Surface tension (mN/m)	Diameter of clear zone (cm)	Overall weight
50	0.52	2.83	45.05	4.65	0.1040
75	0.50	2.47	44.04	4.50	0.1400
100	0.48	3.28	44.15	6.10	0.3870
125	0.40	3.37	42.89	5.75	0.3690
*λ_max_*	4.1341	4.1519	4.1582	4.1263	4.14094
*CI*	0.0447	0.0506	0.0527	0.0421	0.04698
*RI*	0.9	0.9	0.9	0.9	0.9
*CR*	0.0497	0.0563	0.0586	0.0468	0.0522

*λ_max_ = 4.0458, *CI* = 0.0153, *RI* = 0.9, *CR* = 0.017 (*λ_max_*: the maximal eigen value, *CI*: the consistency index, *RI*: the random index, *CR*: the consistency ratio).*

The effect of inoculation amount on four test indices of biosurfactant production from strain 3B-2 was presented in **Table 7**. In this test, the goal was the optimal inoculation amount; the alternatives were different inoculation amounts. The *λ_max_* values of the alternatives to the criteria were 4.15, 4.14, 4.09, and 4.04 (*CR* = 0.0573, 0.0502, 0.0338, and 0.0154, respectively). The *λ_max_* value of the criteria to the goal was 4.05 (*CR* = 0.017), and that of the alternatives to the goal was 4.08 (*CR* = 0.0311). These *CR* values were less than 0.1 and passed the consistency test. The obtained *λ_max_* values were taken as the weights of the test indices to obtain their overall weight. Results showed that within the range tested (4, 6, 8, and 10%), the highest production of biosurfactant occurred with 4% inoculation amount. A larger or smaller amount of inoculation was not favorable for biosurfactant production from strain 3B-2 (**Table 7**).

**Table 7:** Effect of inoculation size on four test indices of biosurfactant production from strain 3B-2.

Inoculum size (%)	Biomass (OD_600_)	Protein concentration (g/L)	Surface tension (mN/m)	Diameter of clear zone (cm)	Overall weight
4	0.60	2.71	38.25	7.00	0.4999
6	0.61	2.58	36.30	6.00	0.3269
8	0.54	2.47	38.65	6.00	0.0935
10	0.46	2.13	38.98	6.15	0.0798
*λ_max_*	4.1547	4.1354	4.0912	4.0417	4.08397
*CI*	0.0516	0.0451	0.0304	0.0139	0.02799
*RI*	0.9	0.9	0.9	0.9	0.9
*CR*	0.0573	0.0502	0.0338	0.0154	0.0311

*λ_max_ = 4.0458, *CI* = 0.0153, *RI* = 0.9, *CR* = 0.017 (*λ_max_*: the maximal eigen value, *CI*: the consistency index, *RI*: the random index, *CR*: the consistency ratio).*

#### Effect of Carbon and Nitrogen Sources

Biosurfactant-producing bacteria generally require large amounts of oxygen (Grishchenkov et al., 2000). Since oil substrates have low capacity to dissolve oxygen, water-soluble substrates are commonly used as the carbon source to facilitate oxygen dissolution. However, some non-saccharide water-soluble substrates (e.g., ethanol) are known to inhibit the fermentative production of biosurfactants (Busscher et al., 1997). In this study, different carbon sources exhibited varying effects on the production of biosurfactant from strain 3B-2 (**Table 8**).

**Table 8:** Effect of carbon sources on four test indices of biosurfactant production from strain 3B-2.

Carbon source	Biomass (OD_600_)	Protein concentration (g/L)	Surface tension (mN/m)	Diameter of clear zone (cm)	Overall weight
Blank	0.85	4.23	35.28	5.50	0.2081
Maltose	1.13	5.66	42.44	6.55	0.1674
Lactose	0.86	4.30	32.40	6.48	0.2917
Sucrose	0.35	1.75	39.08	0.00	0.0506
Xylose	1.10	5.49	42.95	7.30	0.2445
Glucose	0.40	2.00	40.98	0.00	0.0378
*λ_max_*	4.1125	4.0624	4.189	4.0897	6.154
*CI*	0.0532	0.0217	0.0402	0.0235	0.030876
*RI*	1.24	1.24	1.24	1.24	1.24
*CR*	0.0429	0.0175	0.0324	0.0235	0.0249

*λ_max_ = 4.0459, *CI* = 0.0153, *RI* = 0.9, *CR* = 0.0170 (*λ_max_*: the maximal eigen value, *CI*: the consistency index, *RI*: the random index, *CR*: the consistency ratio).*

The formation of clear zone on oil surface is a unique feature of surfactants, which, together with surface tension, provides an indicator to evaluate the performance of biosurfactant-producing bacteria (Youssef et al., 2004). Strain 3B-2 formed no clear zone when grown on sucrose or glucose. That is, this bacterium did not produce biosurfactant when utilizing sucrose or glucose as carbon source. The clear zone was obvious in bacterial culture with maltose, lactose, and xylose as carbon source. Among these, maltose and xylose supported the production of biosurfactant with higher surface tension.

Meanwhile, the four test indices were measured and the optimization of carbon resources was performed using the AHP. The goal was the optimal carbon source; the alternatives were different carbon sources. The *λ_max_* values of the alternatives to the criteria were 4.11, 4.06, 4.19, and 4.09 (*CR* = 0.0429, 0.0175, 0.0324, and 0.0235, respectively). The *λ_max_* value of the criteria to the goal was 4.05 (*CR* = 0.017), and that of the alternatives to the goal was 6.15 (*CR* = 0.0249). The *CR* values were all less than 0.1 and thus passed the consistency test. The obtained *λ_max_* values were taken as weights of test indices to obtain their overall weight. The results showed that the four test indices were highest with lactose as carbon source. Namely, lactose was the optimal carbon source for production of biosurfactant from strain 3B-2.

The synthesis of biosurfactants is closely related to the metabolism of nitrogen resources. Meanwhile, nitrogen resources provide raw materials to produce the protoplasm and other cellular structures. Thus, nitrogen resources have a critical role in microbial growth and biosurfactant production. **Table 9** shows that the organic nitrogen sources were more conducive to bacterial growth than the inorganic nitrogen sources tested. Higher oil displacement efficiency and surface tension were obtained with organic nitrogen sources consistently. These results demonstrated that strain 3B-2 preferentially utilized organic nitrogen sources to produce biosurfactant, with lower production from inorganic nitrogen sources.

**Table 9:** Effect of nitrogen sources on four test indices of biosurfactant production from strain 3B-2.

Nitrogen sources	Biomass (OD_600_)	Protein concentration (g/L)	Surface tension (mN/m)	Diameter of clear zone (cm)	Overall weight
Peptone	0.47	3.72	36.43	6.00	0.1987
Beef extract	0.38	3.19	39.40	5.44	0.1400
Yeast extraction	0.60	3.82	31.00	5.55	0.2213
Soybean meal	0.47	6.55	31.09	3.00	0.1957
Corn meal	0.14	0.13	39.44	0.00	0.0324
(NH_4_)_2_SO_4_	0.05	0.16	34.33	0.00	0.0497
NH_4_NO_3_	0.05	0.14	34.94	0.00	0.0469
NH_4_Cl	0.05	0.04	43.14	0.00	0.0251
NaNO_3_	0.09	0.42	46.30	0.00	0.0243
Urea	0.10	0.39	32.47	0.00	0.0659
*λ_max_*	9.1466	9.4254	9.3003	9.464	10.438
*CI*	0.0183	0.0532	0.0375	0.058	0.0487
*RI*	1.49	1.49	1.49	1.49	1.49
*CR*	0.0126	0.0367	0.0259	0.04	0.0327

*λ_max_ = 4.0458, *CI* = 0.0153, *RI* = 0.9, *CR* = 0.017 (*λ_max_*: the maximal eigen value, *CI*: the consistency index, *RI*: the random index, *CR*: the consistency ratio).*

The AHP was used to determine the optimal nitrogen source for biosurfactant production from strain 3B-2. In this test, the goal was the optimal nitrogen source; the alternatives were different nitrogen sources. The *λ_max_* values of the alternatives to the criteria were 9.15, 9.42, 9.30, and 9.46 (*CR* = 0.0126, 0.0367, 0.0259, and 0.0400, respectively). The *λ_max_* value of the criteria to the goal was 4.05 (*CR* = 0.017) and that of the alternatives to the goal was 10.44 (*CR* = 0.0327). The *CR* values were all less than 0.1 and thus passed the consistency test. The obtained *λ_max_* values were taken as the weights of test indices, from which the overall weight was obtained. The highest production of biosurfactant from strain 3B-2 was obtained using yeast extract as nitrogen source (**Table 9**).

#### Effect of Salinity and Ions

The osmotic pressure of culture solution increases greatly with the increase of NaCl concentration in the medium (Greenway and Osmond, 1972). Microorganisms generally grow well in isotonic solution. Under hypotonic conditions, a large number of water molecules in solution will penetrate into the microbial cells, leading to swelling and rupture; under hypertonic condition, cells will undergo plasmolysis (Marquis, 1968). **Table 10** shows that in the absence of NaCl, strain 3B-2 almost had no growth and its biomass was substantially lower than that in other groups with NaCl. The poor growth led to a low production of biosurfactant from the bacterium. When 30 g/L NaCl was supplied, the diameter of clear zone was zero, and the high salinity might negatively affect the surface tension of biosurfactant produced.

**Table 10:** Effect of salinity concentration on four test indices of biosurfactant production from strain 3B-2.

NaCl (%)	Biomass (OD_600_)	Protein concentration (g/L)	Surface tension (mN/m)	Diameter clear zone (cm)	Overall weight
0	0.06	0.00	48.00	0.00	0.0276
0.5	0.35	2.62	44.07	5.65	0.2139
1	0.46	3.88	45.22	3.75	0.1226
1.5	0.56	3.83	42.27	4.50	0.1758
2	0.60	3.97	37.68	4.70	0.2337
3	0.68	4.01	34.57	0.00	0.2265
*λ_max_*	6.1873	6.104	6.315	6.1497	6.1922
*CI*	0.0375	0.0208	0.063	0.0299	0.03844
*RI*	1.24	1.24	1.24	1.24	1.24
*CR*	0.0302	0.0168	0.0508	0.02241	0.031

*λ_max_ = 4.0458, *CI* = 0.0153, *RI* = 0.9, *CR* = 0.017 (*λ_max_*: the maximal eigen value, *CI*: the consistency index, *RI*: the random index, *CR*: the consistency ratio).*

In this test, the goal was the optimal NaCl concentration; the alternatives were different NaCl concentrations. The *λ_max_* values of the alternatives to the criteria were 6.19, 6.10, 6.32, and 6.15 (*CR* = 0.0302, 0.0168, 0.0508, and 0.0224, respectively). The *λ_max_* value of the criteria to the goal was 4.05 (*CR* = 0.017) and that of the alternatives to the goal was 6.19 (*CR* = 0.031). All the above *CR* values were less than 0.1 and thus passed the consistency test. The obtained *λ_max_* values, i.e., the weights of test indices, were summed up to obtain the overall weight of the four test indices. According to the results, 20 g/L was selected as the optimal NaCl concentration for biosurfactant production from strain 3B-2.

Trace ions are associated with biosurfactant synthesis and serve as the coenzyme of key enzymes involved in the synthetic process. A lack of certain ion(s) or an improper proportional relation of these ions will destroy the entire biosurfactant synthesis system. **Table 11** shows that the majority of the ions tested had an inhibitory effect on biosurfactant production from strain 3B-2. An exception was that Na_2_HPO_4_ to some extent increased biosurfactant production from the bacterium.

**Table 11:** Effect of ion on four test indices of biosurfactant production from strain 3B-2.

Ions	Biomass (OD_600_)	Protein concentration (g/L)	Surface tension (mN/m)	Diameter of clear zone (cm)	Overall weight
Blank	0.59	3.71	37.55	6.00	0.1666
ZnSO_4_	0.33	2.87	32.55	5.25	0.0715
Na_2_HPO_4_	0.66	3.70	29.18	5.95	0.2577
CaCl_2_	0.60	3.81	36.00	5.45	0.1149
BaCl_2_	0.60	3.61	41.85	5.40	0.0752
CuSO_4_	0.32	2.76	32.43	4.50	0.0549
MgSO_4_	0.59	3.47	33.98	4.25	0.0555
MnSO_4_	0.62	3.49	34.16	5.55	0.1004
FeCl_3_	0.60	3.37	33.17	5.50	0.1034
*λ_max_*	9.4254	9.3003	9.464	9.464	9.379
*CI*	0.0183	0.0532	0.0375	0.058	0.0474
*RI*	1.45	1.45	1.45	1.45	1.45
*CR*	0.0126	0.0367	0.0259	0.04	0.0327

*λ_max_ = 4.0458, *CI* = 0.0153, *RI* = 0.9, *CR* = 0.017 (*λ_max_*: the maximal eigen value, *CI*: the consistency index, *RI*: the random index, *CR*: the consistency ratio).*

In this test, the goal was the optimal ion; the alternatives were different NaCl concentrations. The *λ_max_* values of the alternatives to the criteria were 9.43, 9.30, 9.46, and 9.46 (*CR* = 0.0126, 0.0367, 0.0259, and 0.04, respectively). The *λ_max_* value of the criteria to the goal was 4.05 (*CR* = 0.017) and that of the alternatives to the goal was 9.38 (*CR* = 0.0327). All the above *CR* values were less than 0.1 and thus passed the consistency test. According to the overall weight of the *λ_max_* values (e.g., weights), Na_2_HPO_4_ was determined to be the optimal ion species biosurfactant production from strain 3B-2.

#### Optimization of Culture Conditions by CCD

According to the results of the single-factor experiments, lactose, yeast extract, and NaCl were selected as the primary factors to improve biosurfactant production from 3B-2. The experimental design and results are shown in **Table 4**. The four test indices including the biomass, protein concentration, surface tension, and diameter of clear zone were normalized. The lower the surface tension is, the higher the surface activity. Thus, surface tension was set to -1; the other three indices were set to 1. The overall weight of the actual values was obtained from the weights of individual indices presented in Section “Effect of Physical Parameters.”

##### Regression equation and significance analysis

The results of ANOVA analysis for a quadratic response surface model of biosurfactant production are presented in **Table 12**. Model factors *X_2_, X_3_, X_1_X_3_*, and *X_22_* showed extremely significant effects on biosurfactant production from strain 3B-2 (*p* < 0.01). Additionally, an interaction term, *X_2_X_3_*, significantly affected biosurfactant production from the bacterium (0.01 < *p* < 0.05). These results indicated that yeast extract and NaCl concentration had the greatest impact on biosurfactant production from 3B-2, while lactose had the least effect. The *F*-value was 35.35, showing the statistical significance of the model. The effect from other interfering factors on this model was only 0.01%.

**Table 12:** Analysis of variance (ANOVA) for a response surface quadratic model for biosurfactant production of strain 3B-2.

Source	Sum of squares	df	Mean square	*F*	*p* (prob. > *F*)	Significance
Model	4.46E-03	9	4.95E-04	35.35	<0.0001	^∗∗^
*X*_1_-Lactose	1.72E-05	1	1.72E-05	1.23	0.2937	
*X*_2_-Yeast extract	3.28E-03	1	3.28E-03	233.93	<0.0001	^∗∗^
*X*_3_-NaCl	5.46E-04	1	5.46E-04	38.93	<0.0001	^∗∗^
*X*_1_*X*_2_	5.15E-05	1	5.15E-05	3.68	0.0842	
*X*_1_*X*_3_	2.57E-04	1	2.57E-04	18.31	0.0016	^∗^
*X*_2_*X*_3_	8.52E-05	1	8.52E-05	6.08	0.0334	^∗^
*X*_12_	3.26E-07	1	3.26E-07	0.023	0.8819	
*X*_22_	2.23E-04	1	2.23E-04	15.91	0.0026	^∗^
*X*_32_	3.16E-06	1	3.16E-06	0.23	0.6449	
Residual	1.40E-04	10	1.40E-05			
Lack of fit	1.29E-04	5	2.57E-05	11.15	0.0096	^∗∗^
Pure error	1.15E-05	5	2.31E-06			
Cor total	4.60E-03	19				
*R^2^*= 0.9695	R_adj_^2^= 0.9421	R_pred_^2^= 0.7826				

*^∗^*p* < 0.05 (significant); ^∗∗^*p* < 0.01 (highly significant).*

The experimental data were fitted by stepwise regression using Design Expert 8.0. A multivariate quadratic regression model was obtained: *Y* = -0.090205 + 9.13708*E*-003*X_1_* + 0.014698*X_2_* + 2.88754*E* - 003*X_3_* - 4.22917*E* - 004*X_1_X_2_* – 2.83125*E* - 004*X_1_X_3_* - 1.08750*E* - 004*X_2_X_3_* - 3.75750*E* - 005*X_12_* - 4.37036*E* - 004*X_22_* - 4.68498*E* - 006*X_32_*, wherein *Y* is the predicted value of the weight of biosurfactant, and *X_1_* to *X_3_* are lactose, yeast extract, and NaCl, respectively.

For a good statistical model, there is *R^2^* = 0–1.0. A *R^2^* value closer to 0 is indicative a better model and the actual value closer to the predicted value (Reddy et al., 2008). The regression equation obtained by ANOVA showed that the established model had *R^2^* = 0.9695 (*p* < 0.0001). This result indicated that the experimental values of the weight of biosurfactant well agreed with the regression value of the model. Additionally, the model showed high statistical significance. The correction coefficient was: *R^2^*adj = 0.9421; that is, the model could explain 94.21% of variation in the response value. The prediction coefficient was: *R^2^*pre = 0.7826; the lack of fit items was not significant (*F* = 11.15, *p* = 0.096 > 0.05), indicating that the model could fully reflect the actual situation. Collectively, the above data demonstrated good fit of the model. Hence, the proposed model can be used to analyze and predict the effects of lactose, yeast extract and NaCl on biosurfactant production from strain 3B-2 strain.

##### Response surface analysis

**FIGURE 4:**
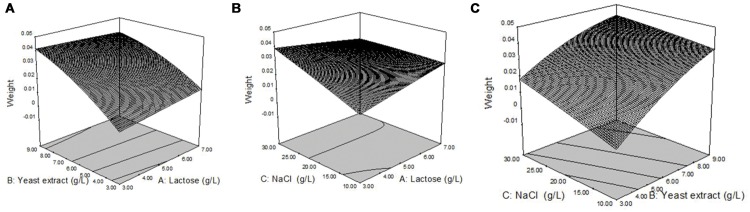
**Response surface plots of biosurfactant production from strain 3B-2. (A)** Interactive effect of lactose and yeast extract with 20 g/L NaCl; **(B)** Interactive effect of lactose and NaCl with 0.6% yeast; **(C)** Interactive effect of yeast extract and NaCl with 0.5% lactose.

Response surface plots of biosurfactant production from strain 3B-2 (**Figure 4**) were generated from the quadratic regression equation. The shape of the response surface was analyze to evaluate the interactive and individual effects of relevant factors on the response value. The contour of the model was nearly horizontal, rather than elliptical, possibly because the interactive effect of relevant factors was relatively weak (Reddy et al., 2008).

In the presence of 20 g/L NaCl, the production of biosurfactant increased with increasing concentrations of lactose and yeast extract, and a greater increase in biosurfactant production was observed with yeast extract (**Figure 4A**). In the presence of 6 g/L yeast extract, lactose, and NaCl concentration changes had little effect on biosurfactant production (**Figure 4B**). In the presence of 5 g/L lactose, biosurfactant production increased with increasing concentrations of NaCl and yeast extract, but the effect of NaCl concentration change on biosurfactant production was less (**Figure 4C**).

### Purification of Crude Biosurfactant

**FIGURE 5:**
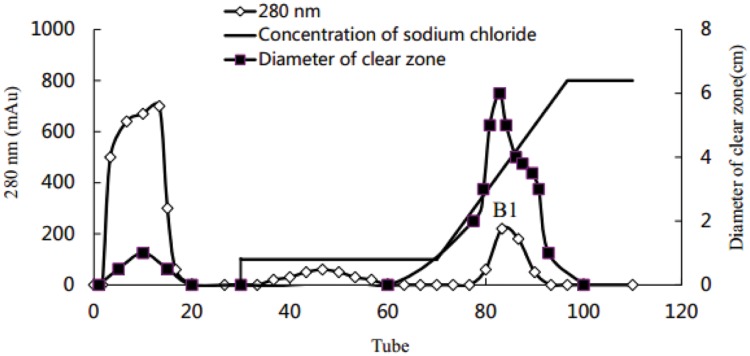
**Purification of crude biosurfactant from strain 3B-2 by DEAE-FF column chromatography**.

**FIGURE 6:**
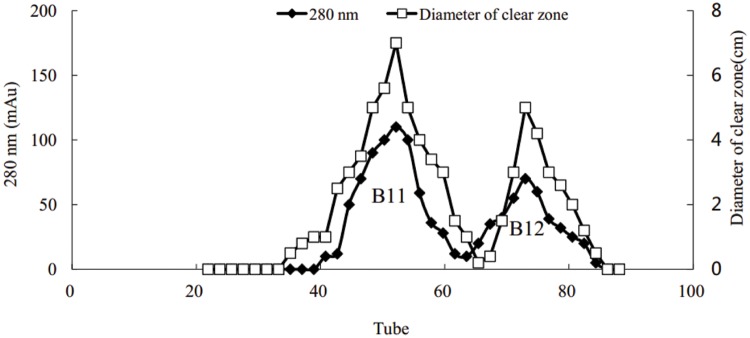
**Purification of biosurfactant fraction B1 from strain 3B-2 by Sephacryl S-100 column chromatography**.

Using the Hiprep DEAE-FF column, protein peaks were obtained at 10, 46, and 81 tube, which exhibited lower activity, no activity, and higher oil-displacement activity, respectively (**Figure 5**). The last eluting peak was named biosurfactant B1. SDS-PAGE analysis showed that the purity of B1 was relatively low. Then B1 was concentrated and purified by gel filtration chromatography on the Sephacryl S-100 column, which resulted in two peaks, named B11 and B12, respectively (**Figure 6**). Oil-displacement test data of B11 and B12 agreed with the experimental results of protein peaks Sephacryl S-100 column.

**FIGURE 7:**
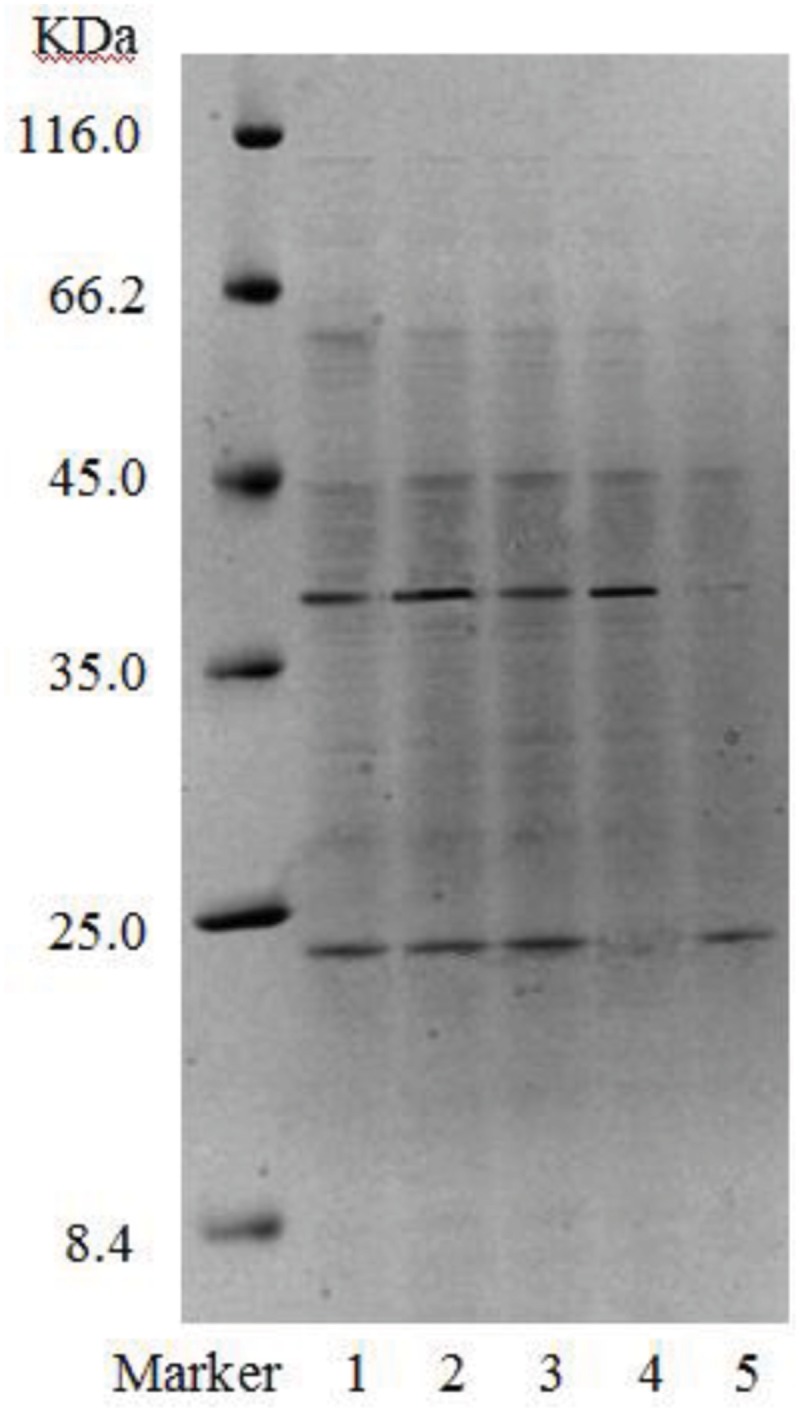
**SDS-PAGE of purified fractions of biosurfactant from strain 3B-2 (Lanes 1–3: samples collected on DEAE-FF column, corresponding to three protein peaks in **Figure 5**; lanes 4 and 5: samples collected on S-100 column, corresponding to two protein peaks in **Figure 6**)**.

Gel electrophoresis revealed that in addition to two strong bands (i.e., B1 and B2), there were other miscellaneous protein bands in the samples collected from the DEAE-FF column (**Figure 7**, lanes 1–3). Clearly, the three samples were not completely purified on the DEAE-FF column. Samples collected from the Sephacryl S-100 column only showed a single clear band each (**Figure 7**, lanes 4 and 5), which was indicative of their high purity. The molecular weights of B11 and B12 were ∼40 and 22 kDa, respectively.

## Conclusion

Biosurfactant production by Marine *Vibrio* sp. strain 3B-2 was evaluated using RSM and AHP techniques to find the optimal composition of growth medium. Experimental results showed that the optimal medium contained: lactose, yeast extract, sodium chloride and disodium hydrogen phosphate according to proportion mentioned above was the best solution. And the optimum growth temperature used in this manuscript was 28°C. The results of this study indicated that biosurfactant can efficiently reduce surface and interfacial tension, and has high oil-displacement efficiency. This finding has a practical advantage because the use of this kind of biosurfactant as a means to promote the degradation of oil may result in wider application of biosurfactant in bioremediation. The features of the biosurfactant production by Marine *Vibrio* sp. strain 3B-2 make it a promising agent for cleaning up environments contaminated with petroleum compounds. The potential of this kind of biosurfactant for bioremediation application is highly dependent on biotic and abiotic parameters, thus further studies to check its activity and ability in hydrocarbon remediation will be recommended.

## Conflict of Interest Statement

The authors declare that the research was conducted in the absence of any commercial or financial relationships that could be construed as a potential conflict of interest.

## Footnotes

1 http://www.ncbi.nlm.nih.gov

2 www.megasoftware.net
